# Treatment of capillary and venous cutaneous vascular malformations using long pulse 1,064-nm neodymium:yttrium-aluminum-garnet laser and intense pulsed light^☆^

**DOI:** 10.1016/j.abd.2023.12.009

**Published:** 2024-08-06

**Authors:** Ludmilla Cardoso Gomes, Mariana Figueiroa Careta, Isabelle I. Hue Wu, Vivian Barzi Loureiro, Luís Antonio Ribeiro Torezan

**Affiliations:** Laser Outpatient Clinic, Hospital das Clínicas, Faculty of Medicine, Universidade de São Paulo, São Paulo, SP, Brazil

Dear Editor,

Cutaneous vascular malformations are anomalies characterized by vessel ectasia, with a tendency to progress. Its predominant location on the face causes aesthetic and psychological harm, with early treatment being indicated in childhood.^1^ Although the Pulsed Dye Laser (PDL) is the reference treatment for these lesions,^2^, ^3^, ^4^ it is not very accessible and its wavelength between 585 and 600 nm hampers treatment of large vessels and those located deeper into the skin. Alternatives such as the long pulse 1,064-nm neodymium:yttrium-aluminum-garnet (Nd:YAG) laser and Intense Pulsed Light (IPL) are used effectively in the treatment of these lesions. Port wine stains (PWS) and capillary vascular malformations are the most studied vascular anomalies in laser therapy.^3^, ^5^

A retrospective study was carried out, approved by the Research Ethics Committee, aimed at evaluating the response of capillary and venous vascular malformations to treatment with the use of IPL and the long-pulse 1,064 nm Nd:YAG laser, in the Dermatology Department of a tertiary hospital. The Free and Informed Consent Form (TCLE) was read, explained, and applied to patients or their legal guardians, as well as the Questionnaire for Global Assessment of the treatment to those who consented to participate in the study.

The participants were divided into two groups, according to the treatments received: (1) IPL (patients treated using IPL alone); (2) Nd:YAG laser +/− IPL (patients treated using the Nd:YAG laser alone or associated with IPL).

The devices used were: IPL ‒ Etherea® (Vydence Medical), Solon® (LMG Lasers), Omnimax® (Sharp Light), and Xeo® (Cutera); and long-pulse Nd:YAG 1,064 nm Laser ‒ Etherea®, Solon®.

Two dermatologists experienced in laser use analyzed the therapeutic response, comparing photographs before and after the treatments, following standardized scores for evaluating lesion clearing^6^: 0 = No response to treatment; 1 = Improvement of 1% to 24% (Slight); 2 = Improvement between 25% and 49% (Moderate); 3 = Improvement between 50% and 74% (Good); 4 = Improvement between 75% and 100% (Excellent). When comparing quantitative variables, the non-parametric Mann-Whitney test was used and the association between qualitative variables was assessed using Fisher's exact test. The significance level adopted was 5% for the statistical tests.

Thirty patients were evaluated, and treated between September 2013 and February 2019. Participants ages ranged from 13 to 74 years old, with an average of 38.4 years. The majority were female (70%) and the most common phototype was phototype III (50%). Treatment with IPL alone was performed in 18 patients, while 12 patients received treatment with Nd:YAG laser associated or not with IPL. Only one case of venous vascular malformation was treated with the long-pulse Nd:YAG laser alone.

According to the evaluation by a dermatologist, all patients achieved improvement in the clearing of the lesions (Fig. 1, Fig. 2). Photographic analysis showed good or excellent response in 66.6% of the patients treated with Nd:YAG laser associated or not with IPL and in 27.8% of those treated with IPL alone (p = 0.196; Fig. 3).

**Fig. 1:**
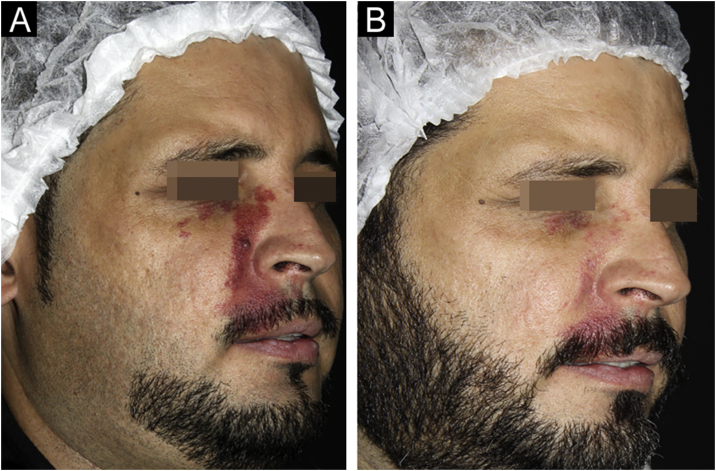
Patient with clearing >75% before (A) and after (B) nine sessions of long-pulse 1,064 nm Nd:YAG laser associated with intense pulsed light.

**Fig. 2:**
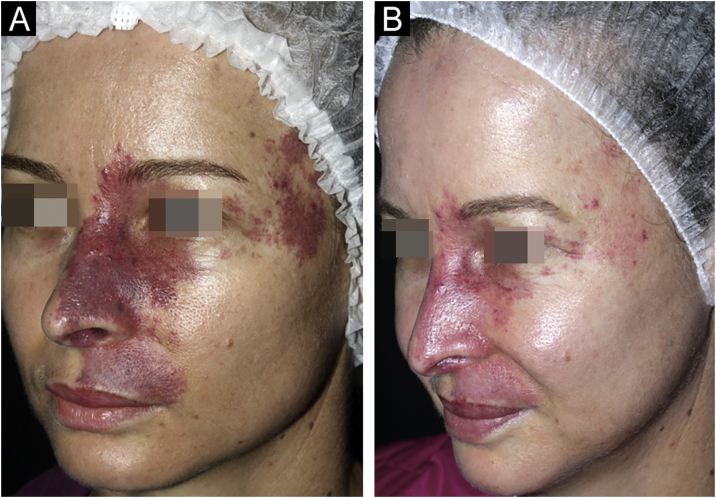
Patient with clearing >75% before (A) and after (B) ten sessions of isolated intense pulsed light.

**Fig. 3:**
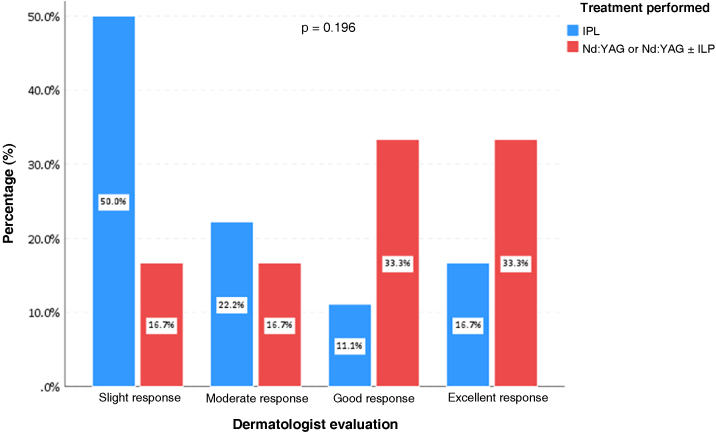
Dermatologist assessment according to the type of treatment performed in patients with capillary and venous cutaneous vascular malformations, São Paulo, 2013 to 2019.

Evaluation by patients based on the questionnaire about their treatment showed improvement in 96.7% of cases. Of these, 56.7% rated the response as good or excellent response regardless of the light source used (p = 0.833). Only one participant (3.3%) did not observe clearing of the lesion with the performed therapy (Fig. 4).

**Fig. 4:**
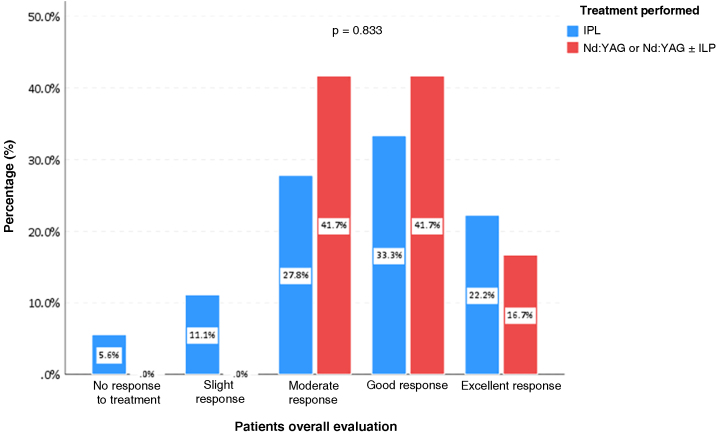
Patient assessment according to the type of treatment performed in patients with capillary and venous cutaneous vascular malformations, São Paulo, 2019 to 2023.

Complications occurred in seven patients (23%). The group treated with IPL alone had three cases (16.7%) of complications (two hyperpigmentation and one hypopigmentation case), *versus* four cases (33.3%) among those treated with Nd:YAG laser associated or not with IPL (one hyperpigmentation, two atrophic scars and one hypertrophic scar; p = 0.392).

Treatment of cutaneous vascular malformations remains a challenge for dermatologists, even after advances in the use of lasers. In 2007, Chapas et al. described the efficacy and safety of treating PWS with PDL in 49 newborns and demonstrated the importance of early treatment for these malformations.^1^ In this study, the authors observed an average age 38.4 years at the start of treatment. This occurrence may be due to the fact that treatments are carried out on an outpatient basis, without general anesthesia or sedation, limiting their application in children.

Liu et al. observed clearing in >90% of the lesions in 20% of patients with capillary vascular malformations resistant to previous use of PDL, treated with long-pulse 1,064 nm Nd:YAG laser.^7^ In the present study, an excellent response was observed in 16.7% and 33.3% in the groups treated with IPL and Nd:YAG laser associated or not with IPL, respectively.

In a review study that investigated the efficacy and adverse effects in patients treated with PDL, IPL and Nd:YAG laser, pain, edema, hypo- and hyperpigmentation, formation of crusts, blisters, hypertrophic scarring and pyogenic granuloma were cited as complications.^5^ Adatto et al. described the safety of using IPL in the treatment of PWS, with transient side effects: pain, erythema, crusts and purpura.^8^ In the present study, the authors observed the occurrence of complications, most of which were temporary (57%). Permanent scars were associated with the 1,064 nm Nd:YAG laser. Avoiding overlapping of laser shots and cooling the skin should be considered to minimize the occurrence of these complications.

Hansen et al. reported satisfaction in 48% of patients, while 28% were neutral and 24% were dissatisfied with the treatment of PWS using PDL.^9^

In this study, 44.4% patients were little satisfied and 55.6% were very satisfied when treated with IPL alone. Among those treated with Nd:YAG laser associated or not with IPL, 16.7% were little satisfied and the majority, 83.3%, were very satisfied with the treatment.

The effectiveness of light sources in the treatment of cutaneous malformations has been widely described in the literature and was evident in the present study, reinforcing the use of the long-pulse 1,064 nm Nd:YAG laser and IPL in venous and capillary cutaneous vascular malformations. However, limitations of this study contributed to the failure to establish statistically significant associations, such as being a retrospective study, subjective analysis, and small sample size.

## Financial support

None declared.

## Authors' contributions

Ludmilla Cardoso Gomes: Approval of the final version of the manuscript; design and planning of the study; drafting and editing of the manuscript; collection, analysis and interpretation of data; intellectual participation in the propaedeutic and/or therapeutic conduct of the studied cases; critical review of the literature; critical review of the manuscript.

Mariana Figueiroa: Collection, analysis and interpretation of data; effective participation in research orientation; intellectual participation in the propaedeutic and/or therapeutic conduct of the studied cases; critical review of the manuscript.

Isabelle I Hue Wu: Approval of the final version of the manuscript; intellectual participation in the propaedeutic and/or therapeutic conduct of the studied cases; critical review of the manuscript.

Vivian Barzi Loureiro: Approval of the final version of the manuscript; intellectual participation in the propaedeutic and/or therapeutic conduct of the studied cases; critical review of the literature.

Luis Antonio Ribeiro Torezan: Statistical analysis; approval of the final version of the manuscript; design and planning of the study; collection, analysis and interpretation of data; effective participation in research orientation; intellectual participation in the propaedeutic and/or therapeutic conduct of the studied cases; critical review of the manuscript.

## Conflicts of interest

None declared.
